# Supplementary material for: Functional Ex Vivo Tissue-Based Chemotherapy Sensitivity Testing for Breast Cancer
Source: Cancers (Basel). 2022 Feb 28;14(5):1252. doi: 10.3390/cancers14051252 (PMC8909506; doi:10.3390/cancers14051252)
Supplement: Supplementary file 1 [file cancers-14-01252-s001.zip › cancers-1584655-supplementary.pdf]

cancers

MDPI

Article

Functional Ex Vivo tissue-based chemotherapy sensitivity testing for breast cancer

Marjolijn M. Ladan, Titia G. Meijer, Nicole S. Verkaik, Zofia M. Komar, Carolien H.M. van Deurzen, Michael A. den Bakker, Roland Kanaar, Dik C. van Gent and Agnes Jager

Table S1. Characteristics of 13 primary breast cancer samples for cisplatin treatment.

Tumor Code	Ex vivo Sensitivity	Receptor Status	Histology	Grade	BRCA Status
M141	Intermediate	ER/PR +, HER2-	ductal	3	Unknown
M143	Intermediate	ER/PR +, HER2-	ductal	3	Unknown
M153	Resistant	ER/PR +, HER2-	ductal	3	Unknown
M167	Intermediate	ER/PR +, HER2-	ductal	3	Non-BRCA
M188	Intermediate	ER/PR +, HER2-	ductal	3	BRCA2 mutation
M335	Intermediate	ER/PR +, HER2-	Neuroendocrine	3	Not tested
M348	Intermediate	HER2+	Mucinous	3	Non-BRCA
M356	Resistant	ER/PR +, HER2-	Ductal	2	Not tested
M357	Resistant	ER/PR +, HER2-	Micropapillar/ductal	2	Unknown
M370	Intermediate	ER/PR +, HER2-	Ductal	1	Unknown
M371	Intermediate	ER/PR +, HER2-	Lobular	2	Unknown
M377	Resistant	ER/PR +, HER2-	Ductal	3	Not tested
M378	Resistant	ER/PR +, HER2-	Ductal	3	Not tested

BRCA status unknown: it is not known whether this patient underwent *BRCA* testing. BRCA status not tested: based on family history and age, there was no necessity to test for *BRCA* mutations in this patient. Non-BRCA: genetic *BRCA* testing was performed, but no pathogenic *BRCA* mutations were found.

Table S2. Characteristics of 10 primary breast cancer tumors for docetaxel treatment.

Tumor Code	Ex vivo Sensitivity	Receptor Status	Histology	Grade
M377	Sensitive	ER/PR +, HER2-	Ductal	3
M382	Sensitive	ER/PR +, HER2-	Ductal	3
M395	Resistant	ER/PR +, HER2-	Ductal	2
M403	Intermediate	ER/PR +, HER2-	Ductal	2
M412	Sensitive	ER/PR +, HER2-	Lobular	2
M424	Resistant	ER/PR +, HER2-	Lobular	3
M425	Intermediate	ER/PR +, HER2-	Lobular	2
M448	Sensitive	ER/PR +, HER2-	Ductal	2
M450	Intermediate	ER/PR +, HER2-	Ductal	2
M459	Sensitive	ER/PR +, HER2-	Lobular	2

Table S3. Characteristics of 20 metastatic biopsies for cisplatin treatment.

Biopsy no	Successful test	Ex vivo sensitivity	Receptor status	Histology	Grade	Biopsy type	Biopsy size	Origin	BRCA status
M238	Yes	Resistant	ER/PR +, HER2-	ductal	3	needle	14G	mamma	Non BRCA
M256	Yes	Intermediate	ER/PR +, HER2-	ductal	3	needle	14G	chestwall	Non BRCA
M290	Yes	Resistant	ER/PR +, HER2-	ductal	3	punch	4mm	chestwall	Non BRCA
M313	Yes	Sensitive	ER/PR +, HER2-	unknown	unknown	needle	18G	liver	Not tested
M363	Yes	Intermediate	ER/PR +, HER2-	ductal	2	needle	18G	LN	Non BRCA
M341	Yes	Intermediate	ER/PR +, HER2-	ductal	3	needle	unknown	LN	BRCA2 mutation
M254	Yes	Resistant	ER/PR +, HER2+	lobular	unknown	needle	14G	LN	Not tested
M265	Yes	Sensitive	TN	ductal	2	needle	14G	mamma	Non BRCA
M350	Yes	Intermediate	TN	ductal	3	needle	14G	mamma	Non BRCA
M366	Yes	Intermediate	TN	ductal	3	needle	14G	mamma	Non BRCA
M303#	Yes	Intermediate	TN	ductal	3	needle	18G	other	BRCA1 mutation, with secondary reversion mutation
M367#	Yes	Intermediate	TN	ductal	3	needle	18G	chestwall	BRCA1 mutation, with secondary reversion mutation
M298	Yes	Resistant	unknown	ductal	3	needle	18G	chestwall	BRCA2 mutation
M227	Partly	Unknown	ER+/PR-/HER2-	ductal	2	needle	18G	liver	Not tested
M234	Party	Unknown	TN	ductal	2	needle	18G	liver	BRCA2 mutation
M212	No		ER/PR +, HER2-	ductal	unknown	needle	18G	liver	Non BRCA
M226	No		ER/PR +, HER2-	ductal	2	needle	18G	liver	Not tested
M362	No		ER/PR+, HER2-	ductal	3	needle	18G	liver	BRCA2 mutation
M294	No		ER/PR +, HER2-	ductal	3	needle	18G	LN	Non BRCA
M308	No		TN	ductal	3	needle	18G	chestwall	Non BRCA
M323	No		TN	ductal	3	needle	18G	chestwall	Non BRCA

# Indicates matching biopsies from the same patient, taken at different time points during disease progression. BRCA status unknown: it is not known whether this patient underwent *BRCA* testing. BRCA status not tested: based on family history and age, there was no necessity to test for *BRCA* mutations in this patient. Non-BRCA: genetic *BRCA* testing was performed, but no pathogenic *BRCA* mutations were found.

Table S4. Comparison of histopathological characteristics of successful tests and non-successful drug sensitivity tests on biopsies.

Tumor characteristic	Non successful test result	Successful test result	P-value
**Histological grade (primary tumor)**			
1	-	-	
2	3	2	
3	4	8	
Unknown	1	2	0.811
**Receptor Status**			
ER/PR +, HER2-	5	6	
HER2+*	-	1	
TNBC	3	5	
Unknown	-	-	1.000
**Biopsy size**			
18 G	8	6	
14 G	-	4	
4 mm	-	1	
Unknown	-	1	0.012
**Metastatic site**			
Breast	-	4	
Chestwall	2	3	
Axillary/Cervical/ Pectoral LN	1	3	
Liver	5	1	
Other	-	1	0.078
BRCA			
Mutation	2	3	
Wild-type	6	9	0.693
**Total**	8	12	

*Independent of ER/PR status. Fisher's exact test. ER/PR+ was defined as >10% ER+ and/or >10% PR+. HER2+ defined as immunohistochemistry (IH) 3+ or IH 2+ and HER2 amplification detected by in situ hybridization.

Table S5. Comparison of histopathological characteristics of *ex vivo* sensitive, intermediate and resistant metastatic biopsies.

Tumor characteristic	Sensitive	Intermediate	Resistant	P-value
**Histological grade (primary tumor)**				
1	-	-	-	
2	1	1	-	
3	-	6	2	
Unknown	1	-	1	0.223
**Receptor Status**				
ER/PR +, HER2-	1	3	2	
HER2+*	-	-	1	
TNBC	1	4	-	
Unknown	-	-	-	0.427
**Biopsy size**				
18 G	1	3	-	
14 G	1	3	2	
4 mm	-	-	1	
Unknown	-	1	-	0.009
**Metastatic site**				
Breast	1	2	1	
Chestwall	-	2	1	
Axillary/Cervical/ Pectoral LN	-	2	1	
Liver	1	-	-	
Other	-	1	-	0.148
BRCA				
Mutation	-	3	0	
Wild-type	2	4	3	0.518
**Total**	2	7	3	

*Independent of ER/PR status. Fisher's exact test. ER/PR+ was defined as >10% ER+ and/or >10% PR+. HER2+ defined as immunohistochemistry (IH) 3+ or IH 2+ and HER2 amplification detected by in situ hybridization.

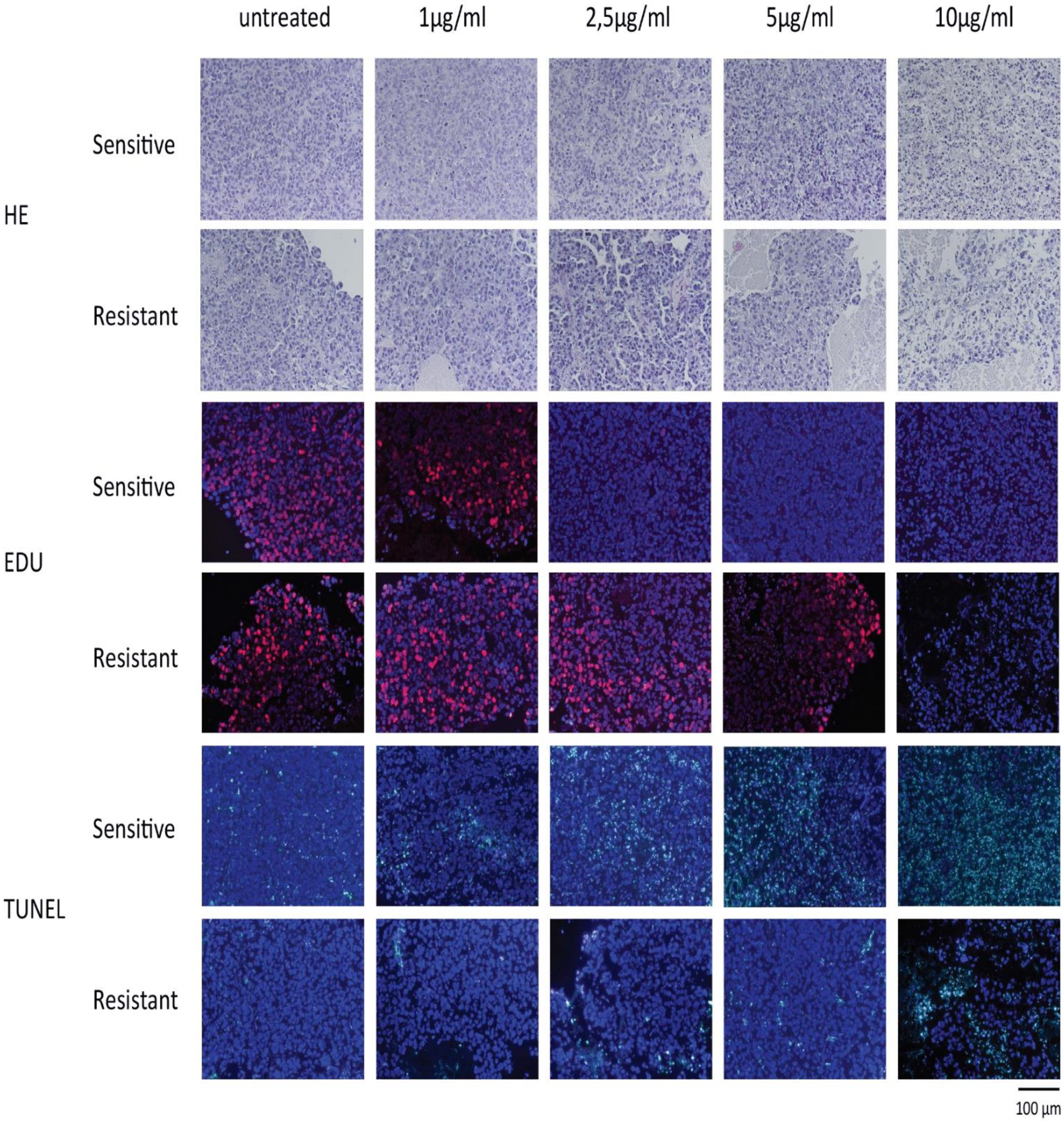

Figure S1. *In* vivo sensitive and resistant PDX tumors show differential response to ex vivo cisplatin treatment. Organotypic tissue slices form a sensitive and a resistant PDX tumor were subjected to ex vivo cisplatin treatment for 3 days. Representative HE, EdU (proliferation) and TUNEL (apoptosis) images are shown for each treatment condition (blue = DAPI, red = EdU, green = TUNEL, magnification x200).

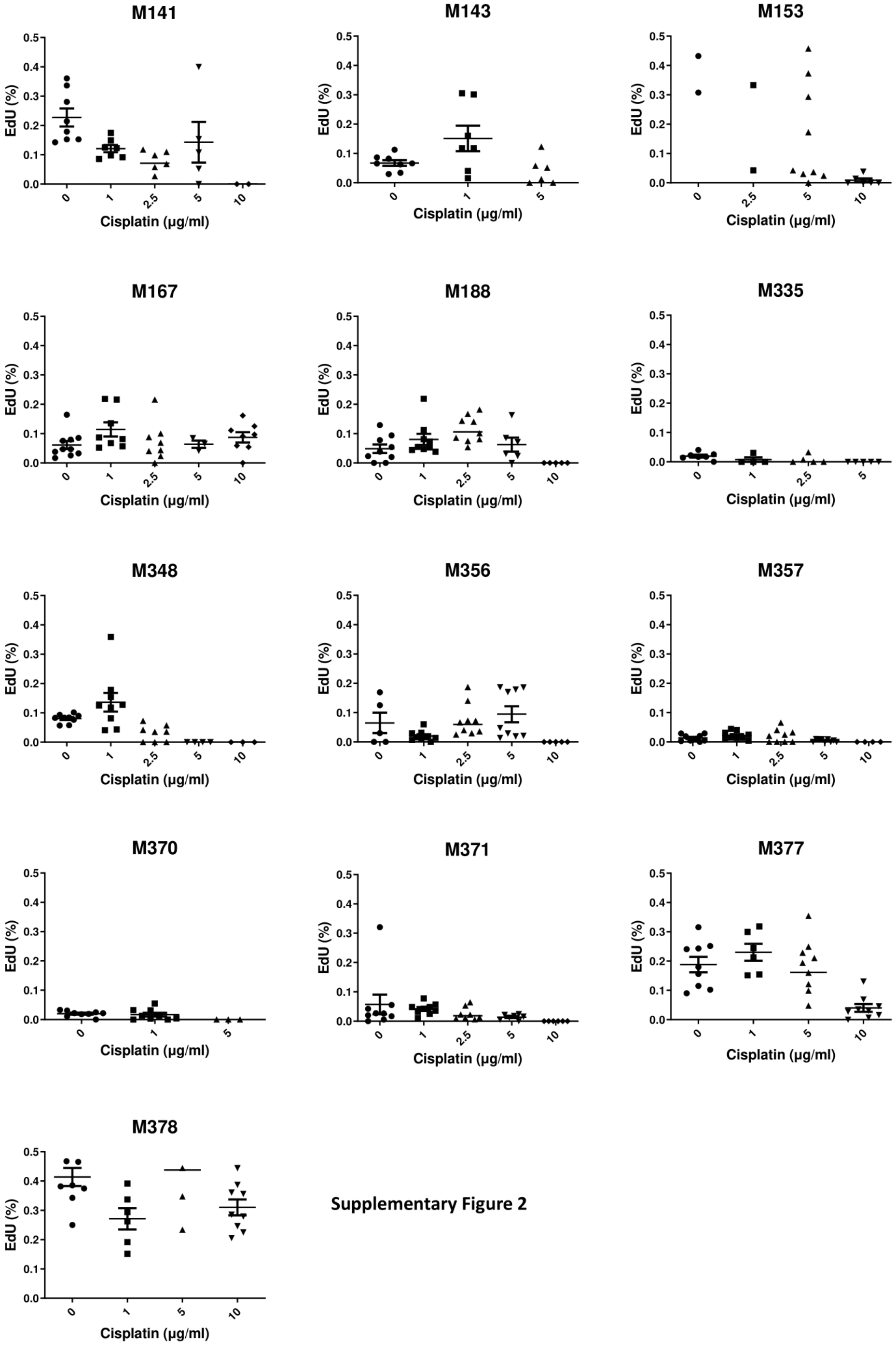

Figure S2. Quantifications of proliferation (EdU incorporation) in primary breast cancer tissue slices. Between three and twelve microscopic fields of view were analyzed per tumor slice. The graphs show each point (each circle, triangle and square representing one field of view) with mean and SEM.

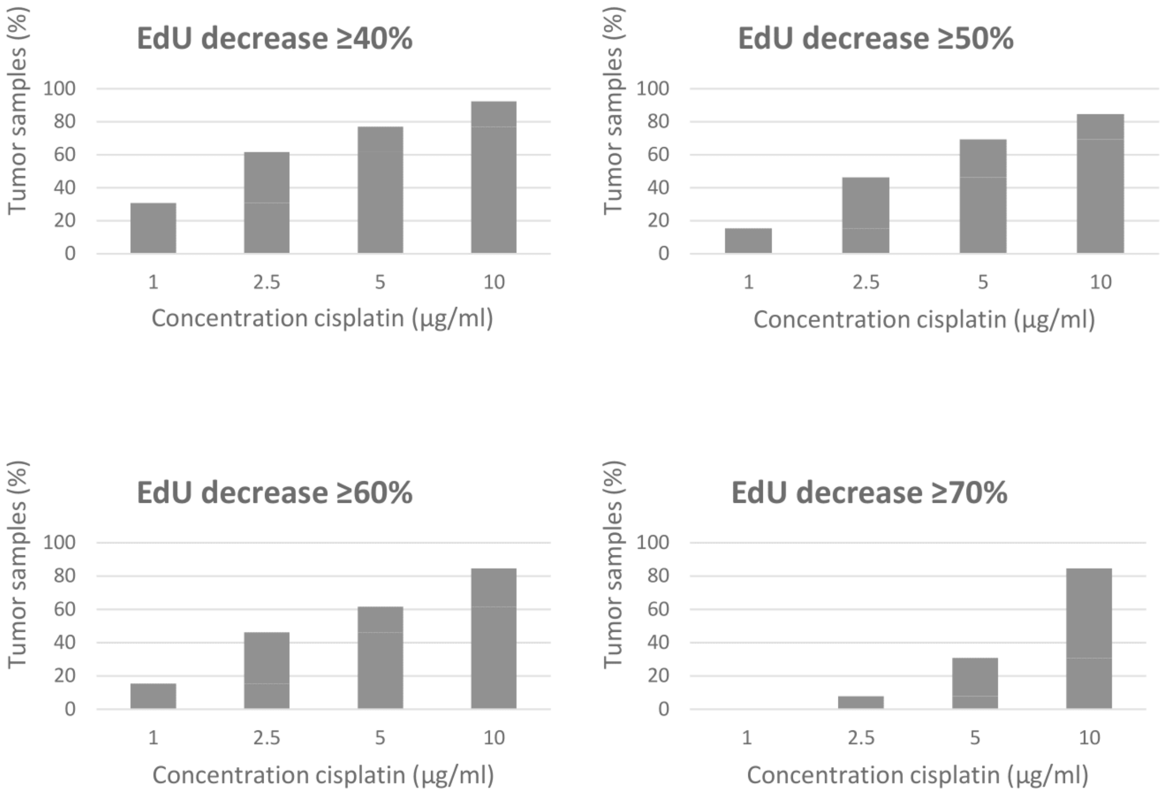

Figure S3. Proliferation based on EdU incorporation relative to untreated primary breast cancer slices (n = 13). Tumor samples (%) scores the cumulative percentage of tumor samples that reached the threshold at or below that concentration.

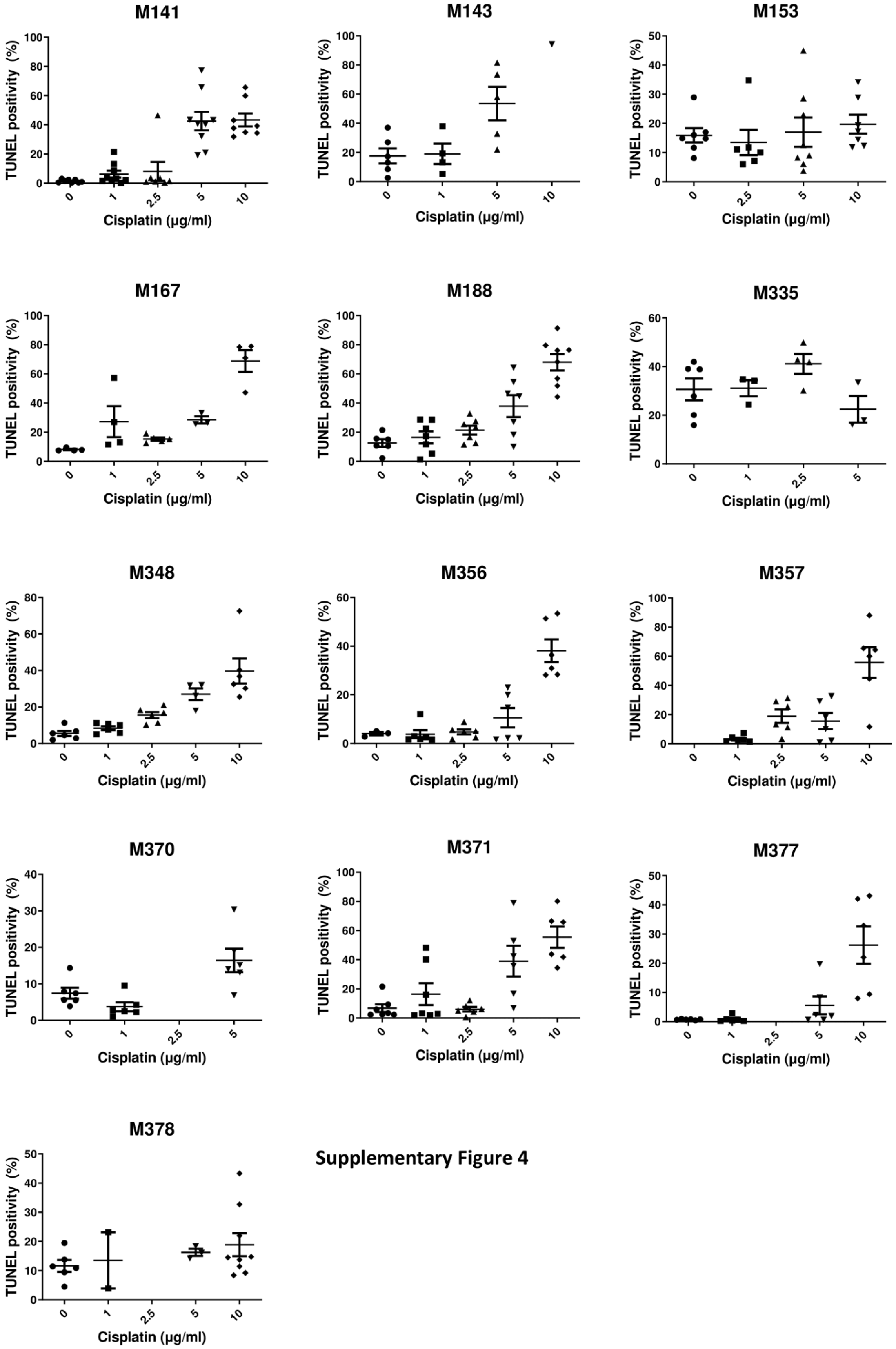

Figure S4. Quantification of apoptosis in primary breast cancer slices. The percentage of TUNEL positive pixels relative to the total number of DAPI positive pixels. Between three and twelve microscopic fields of view were analyzed per tumor slice. The graphs show each point (each circle, triangle and square representing one field of view) with mean and SEM.

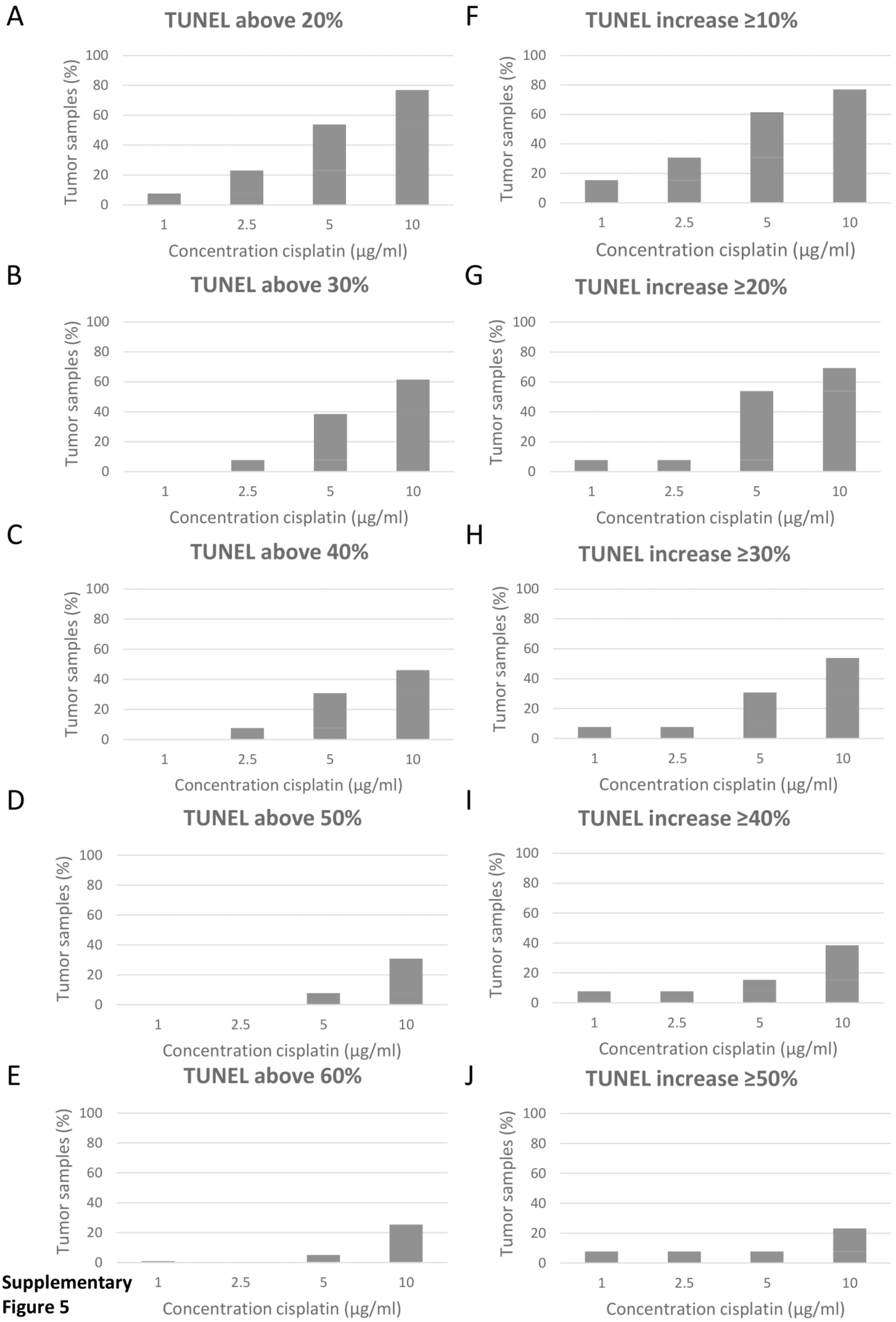

Supplementary Figure 5

Figure S5. Apoptosis measurement using different cut-off values for TUNEL positivity (n = 13). (**A-E**) An absolute value for the cut-off of TUNEL positivity ranging from 20% to 60%, or (**F-J**) an increase of TUNEL compared to the untreated slices, ranging from 10% to 50% increase. Tumor samples (%) scores the cumulative percentage of tumor samples that reached the threshold at or below that concentration.

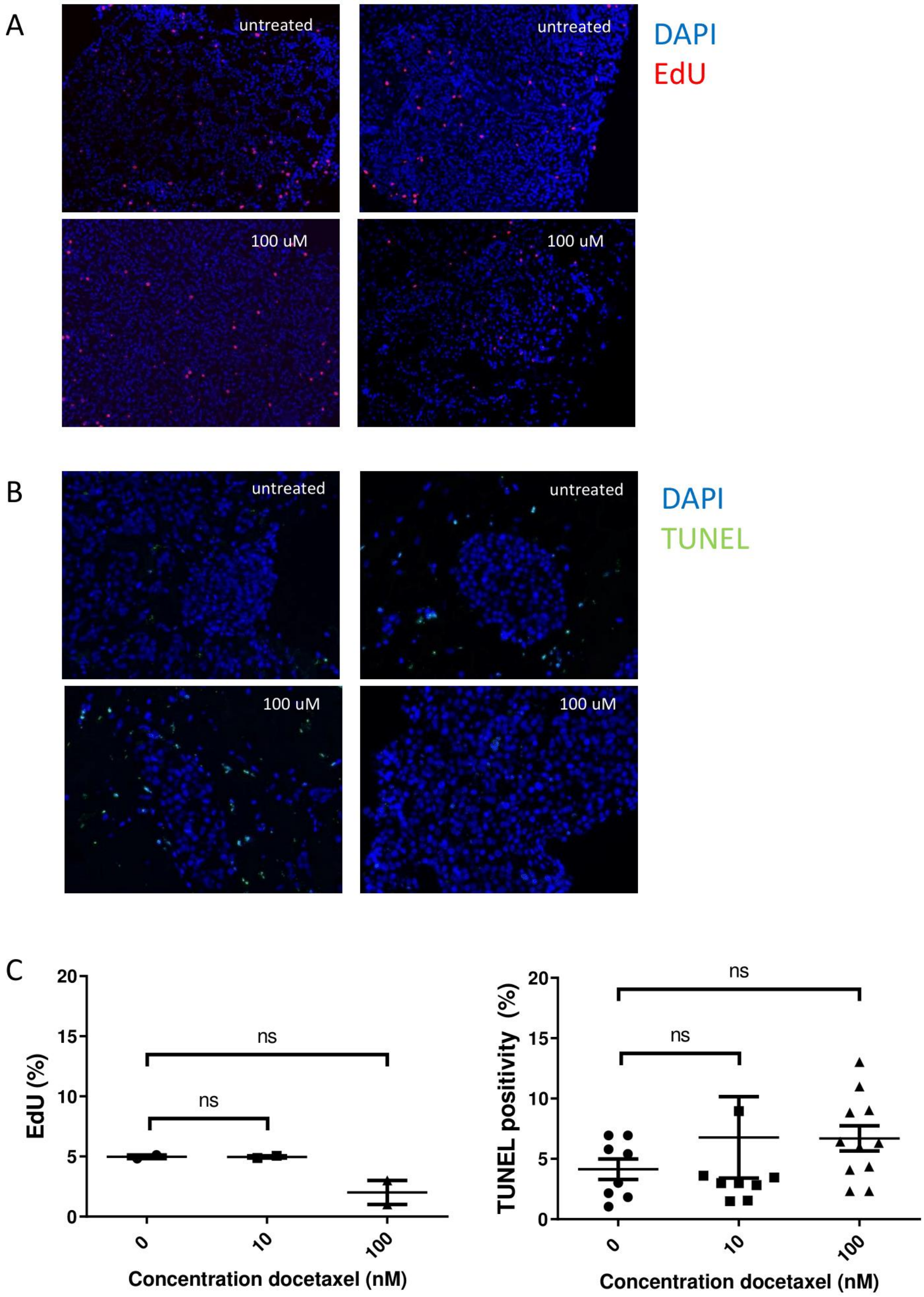

Figure S6. Proliferation and apoptosis after docetaxel treatment. (**A**) Proliferation was measured by EdU staining of primary BC slices treated with 0 and 100 nM docetaxel after 3 days (left panel) and 7 days (right panel). (**B**) Apoptosis was measured by TUNEL staining of primary BC slices treated with 0 and 100 nM docetaxel after 3 days (left panel) and 8 days (right panel). (**C**) Quantification of EdU staining for 0, 10 nM and 100 nM docetaxel on day 3 (left panel) and TUNEL staining for 0, 10 nM and 100 nM docetaxel on day 3 (right panel). The graphs show each point (each circle, triangle and square representing one image field) with mean and SEM. * $P<0.05$, ns = non-significant.

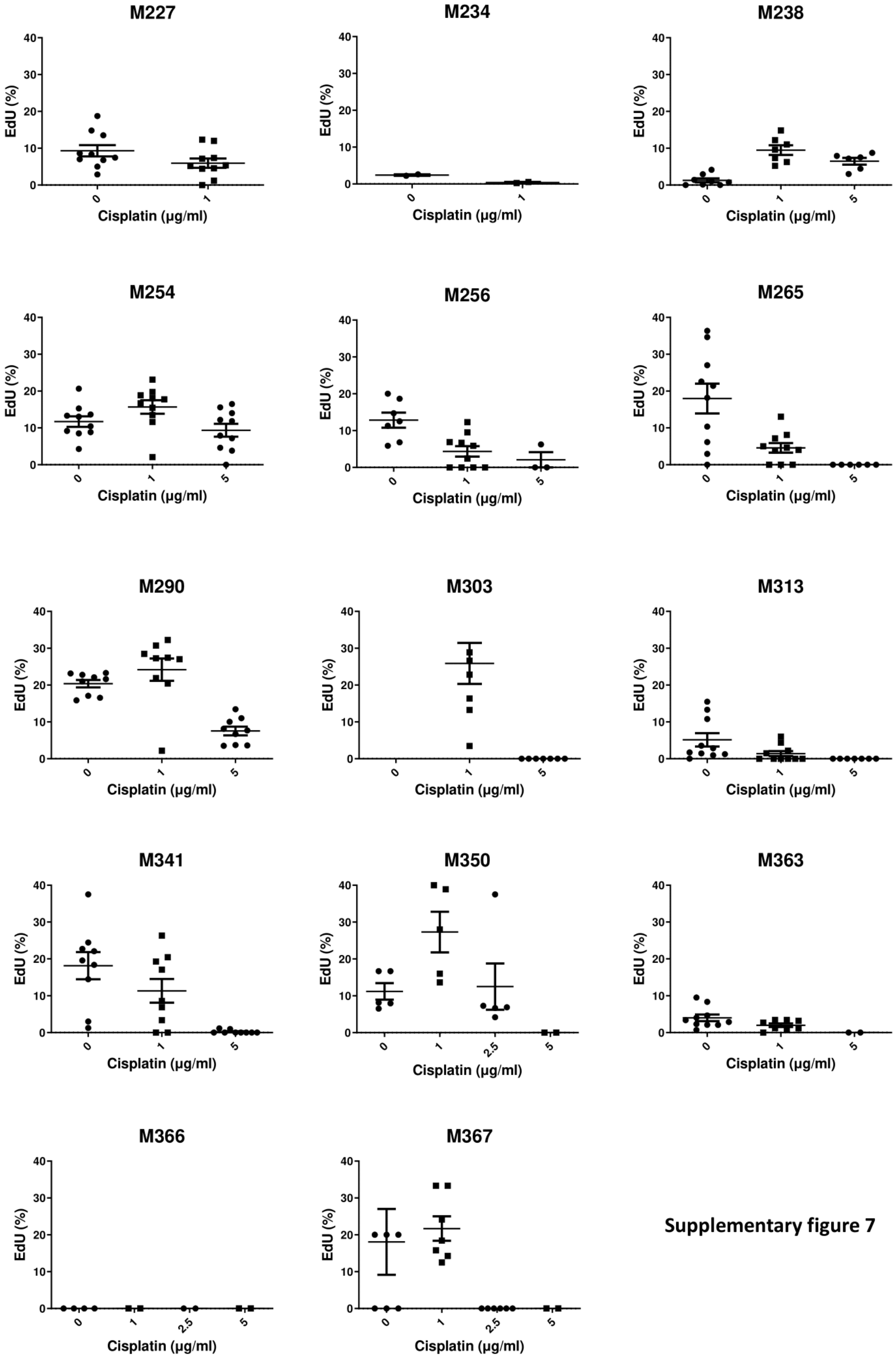

Figure S7. Quantifications of proliferation (EdU incorporation) in metastatic breast cancer biopsy slices. Between three and twelve microscopic fields of view were analyzed per tumor slice. The graphs show each point (each circle, triangle and square representing one field of view) with mean and SEM.

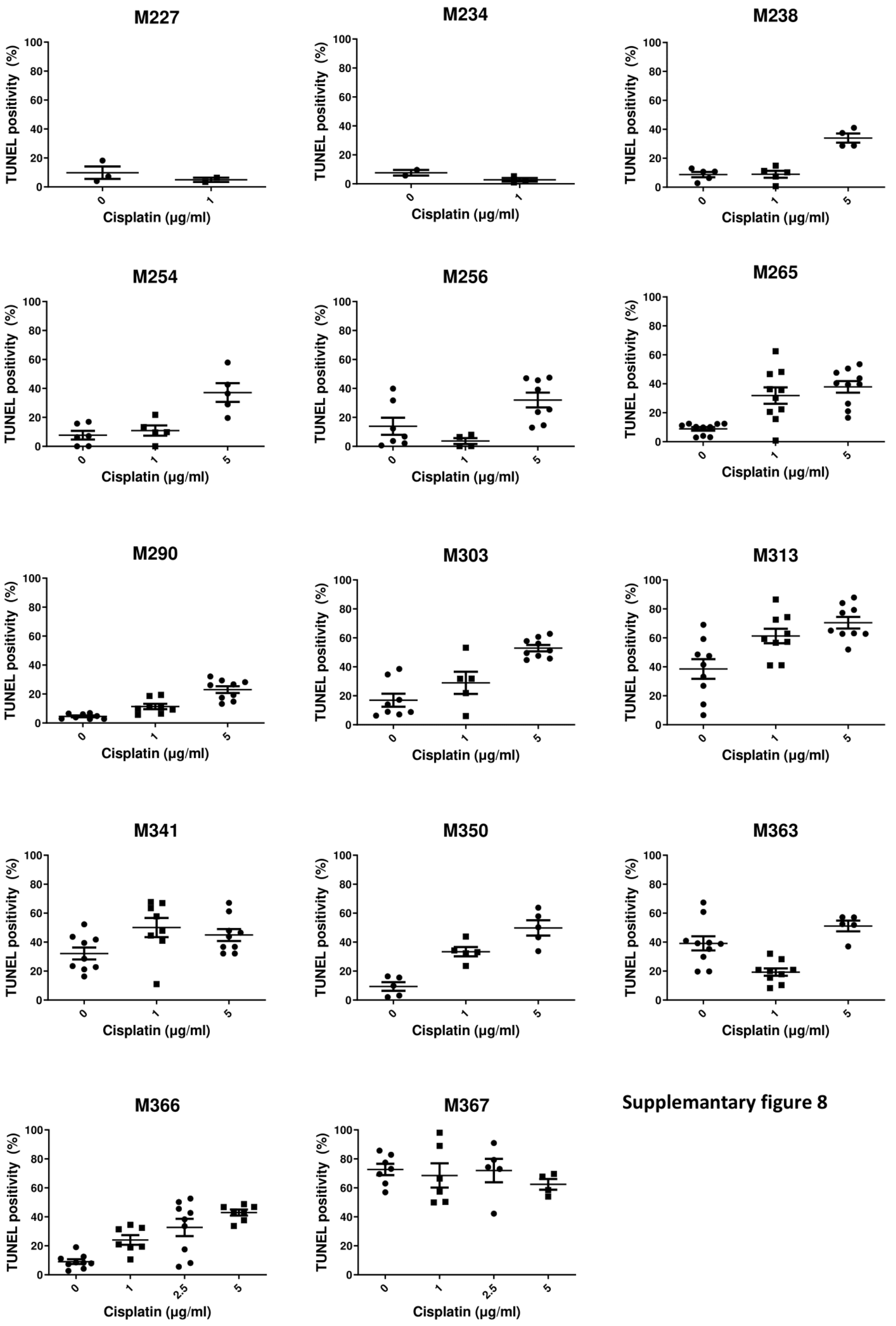

Figure S8. Quantifications of apoptosis (TUNEL staining) in metastatic breast cancer biopsy slices. Between three and twelve fields were analyzed per tumor slice. The graphs show each point (each circle, triangle and square representing one field of view) with mean and SEM.
